# Fully automated assessment of the future liver remnant in a blood-free setting via CT before major hepatectomy via deep learning

**DOI:** 10.1186/s13244-024-01724-6

**Published:** 2024-06-27

**Authors:** Tingting Xie, Jingyu Zhou, Xiaodong Zhang, Yaofeng Zhang, Xiaoying Wang, Yongbin Li, Guanxun Cheng

**Affiliations:** 1 Medical Imaging Center, Peking University Shenzhen Hospital, Shenzhen, Guangdong 518036 China; 2 Department of Radiology, Peking University First Hospital, Beijing, 100034 China; 3 Beijing Smart Tree Medical Technology Co. Ltd, Beijing, China; 4 Department of Ultrasound, Peking University Shenzhen Hospital, Shenzhen, Guangdong 518036 China

**Keywords:** Future liver remnant, Major hepatectomy, Hepatic vein, Portal vein, Deep learning

## Abstract

**Objectives:**

To develop and validate a deep learning (DL) model for automated segmentation of hepatic and portal veins, and apply the model in blood-free future liver remnant (FLR) assessments via CT before major hepatectomy.

**Methods:**

3-dimensional 3D U-Net models were developed for the automatic segmentation of hepatic veins and portal veins on contrast-enhanced CT images. A total of 170 patients treated from January 2018 to March 2019 were included. 3D U-Net models were trained and tested under various liver conditions. The Dice similarity coefficient (DSC) and volumetric similarity (VS) were used to evaluate the segmentation accuracy. The use of quantitative volumetry for evaluating resection was compared between blood-filled and blood-free settings and between manual and automated segmentation.

**Results:**

The DSC values in the test dataset for hepatic veins and portal veins were 0.66 ± 0.08 (95% CI: (0.65, 0.68)) and 0.67 ± 0.07 (95% CI: (0.66, 0.69)), the VS values were 0.80 ± 0.10 (95% CI: (0.79, 0.84)) and 0.74 ± 0.08 (95% CI: (0.73, 0.76)), respectively No significant differences in FLR, FLR% assessments, or the percentage of major hepatectomy patients were noted between the blood-filled and blood-free settings (*p* = 0.67, 0.59 and 0.99 for manual methods, *p* = 0.66, 0.99 and 0.99 for automated methods, respectively) according to the use of manual and automated segmentation methods.

**Conclusion:**

Fully automated segmentation of hepatic veins and portal veins and FLR assessment via blood-free CT before major hepatectomy are accurate and applicable in clinical cases involving the use of DL.

**Critical relevance statement:**

Our fully automatic models could segment hepatic veins, portal veins, and future liver remnant in blood-free setting on CT images before major hepatectomy with reliable outcomes.

**Key Points:**

- Fully automatic segmentation of hepatic veins and portal veins was feasible in clinical practice.
- Fully automatic volumetry of future liver remnant (FLR)% in a blood-free setting was robust.
- No significant differences in FLR% assessments were noted between the blood-filled and blood-free settings.

**Graphical Abstract:**

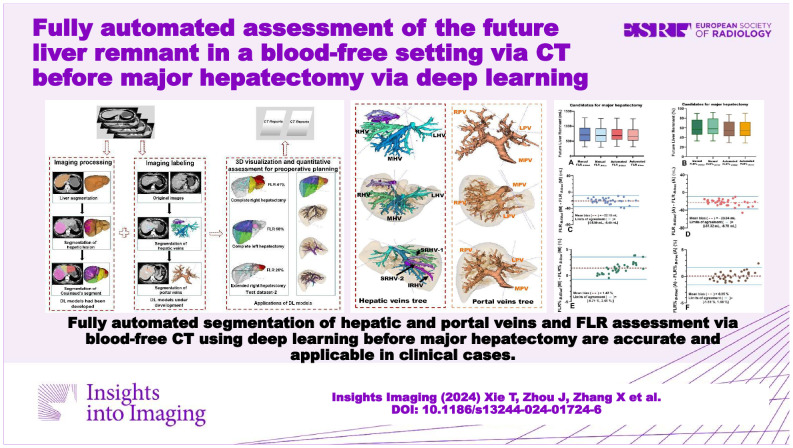

## Background

Post-hepatectomy liver failure (PHLF) is regarded as the primary factor contributing to mortality after major hepatectomy [1–3]. Its prevalence is variable and up to 12–34% [4]. The future liver remnant (FLR) volume is considered one of the most important predictors of PHLF [5], and CT volumetry of the FLR has become an essential procedure before major hepatectomy in clinical practice. However, preoperative CT volumetry has been criticized for under- and overestimating real volumes for several reasons, including (i) the preoperative assumed planes used for volumetry differ from the actual resection planes and (ii) the blood volume contained in the large hepatic vessels (*V*_*blood*_, i.e., the volume of portal veins and hepatic veins) in the graft contributes to the difference between CT volumetry and real liver grafts because volumetry on CT images is blood-filled while intraoperative measurements are blood-free [4, 6, 7]. Since the blood pool comprises more than 9% of the whole liver volume [8], *V*_*blood*_ should be taken into account. Considering that the minimum FLR in normal and diseased liver tissue (i.e., steatosis, cholestasis, and cirrhosis) ranges from 20% to 40%, it is worthwhile to segment and volumeter the hepatic veins and portal veins and to investigate how much blood content contributes to the error of preoperative CT volumetry. Therefore, segmentation and volumetry of hepatic veins and portal veins on CT images, precise assessment of preoperative FLR in a blood-free setting, and comparison of FLR between blood-filled setting and blood-free setting (i.e., FLR _*B-filled*_ and FLR _*B-free*_) are essential.

Several studies have reported the use of deep learning (DL) algorithms for volumetry of the right lobe in living donor liver transplantation; however, the difference in the volumetry of the FLR _*B-filled*_ and FLR _*B-free*_ has not been investigated [9–13]. Moreover, these studies aimed to apply DL algorithms in the preoperative planning of living donor liver transplantation; however, DL models for preoperative FLR assessment of major hepatectomy have rarely been reported and remain unknown.

Several authors have developed deep learning (DL) models for the automated segmentation of hepatic veins and portal veins, which can potentially be used in preoperative FLR assessment prior to major hepatectomy in a blood-free setting; however, these studies have focused on the technical feasibility of developing new DL models to improve segmentation performance, and external validation via the use of various pathologic livers has been ignored [14–17]. How these models perform in real clinical cases, especially under highly variable and complex liver conditions, has not been determined. Severely deformed liver tissue caused by cirrhosis and vascular invasion caused by hepatic tumors can lead to smaller and blur veins than in a healthy liver, and segmentation of hepatic vessels is a major challenge. Almost all these studies mentioned that DL models of hepatic veins and portal veins assist in the planning of hepatic resection [14–17]; however, how these DL models perform on these pathological livers during real preoperative planning has not been fully evaluated.

Therefore, we aimed to develop a DL model for the automatic segmentation and volumetry of hepatic veins and portal veins, validate the models in an external validation cohort with various liver conditions, and apply the model in combination with preoperative FLR _*B-filled*_ and FLR _*B-free*_ assessment prior to major hepatectomy.

## Materials and methods

### Dataset

The training dataset and test dataset were used for the development of a DL model for automated segmentation of Couinaud’s liver segment in a previous study [18].

**Fig. 1:**
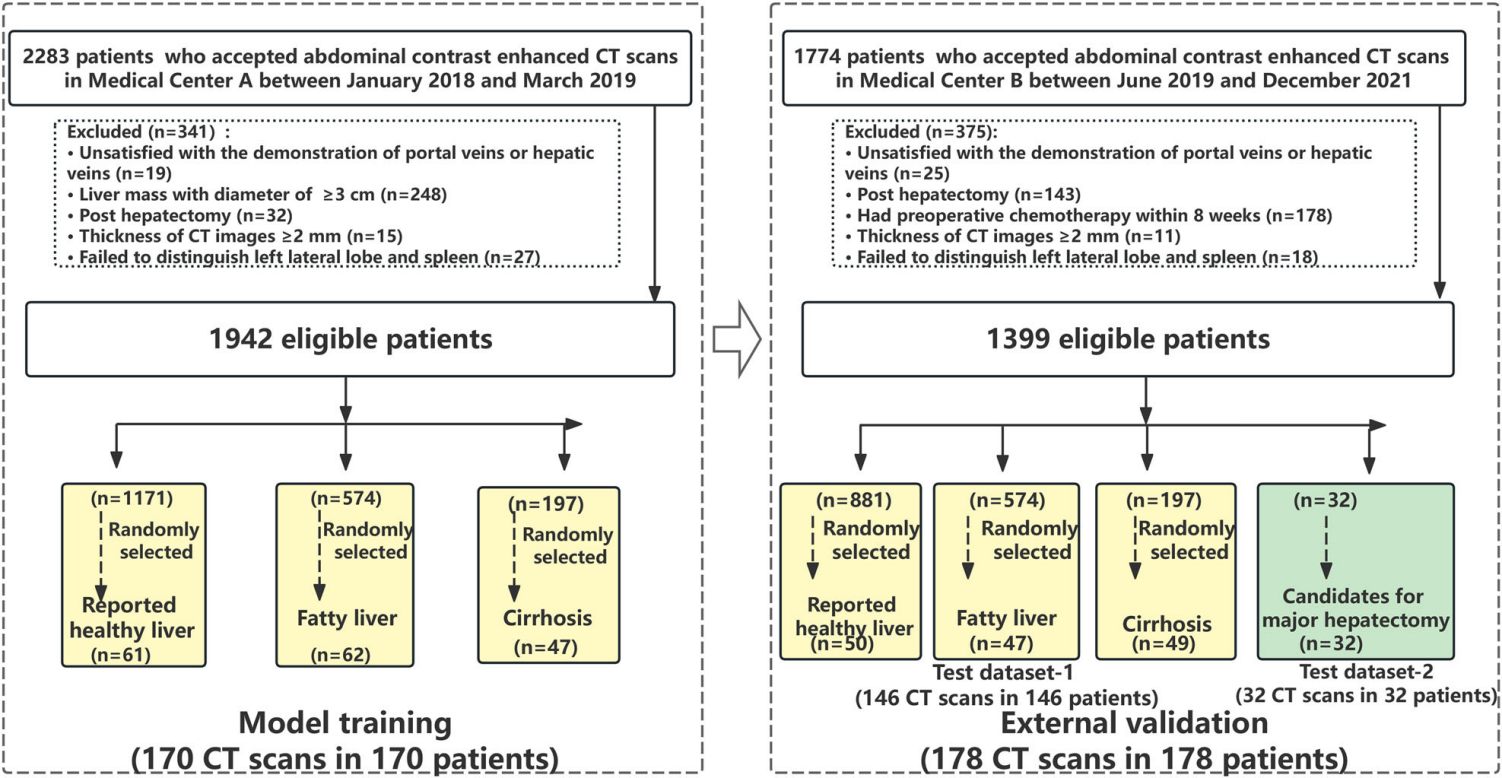
The inclusion criteria, exclusion criteria, and distribution of computed tomography (CT) scans in the data sets used in this study were demonstrated in flowchart. TACE, transcatheter arterial chemo-embolization

The training dataset was extracted from 2283 consecutive patients who underwent liver contrast-enhanced CT scans at Medical Center A (Peking University First Hospital) between January 2018 and March 2019. A total of 170 patients were included in the training dataset cohort. A flowchart is presented in Fig. 1.

Two test datasets were used for the external validation of the DL models. These test datasets were extracted from 1774 consecutive patients who underwent liver contrast-enhanced CT scans at Medical Center B (Peking University Shenzhen Hospital) between June 2019 and December 2021. A total of 178 patients were included.

To develop a robust DL model, CT data extracted from patients with various liver pathologies and obtained by different CT manufacturers were included. The various liver pathologies included fatty liver disease secondary to systemic chemotherapy, alcoholic fatty liver disease, alcohol-associated cirrhosis, nonalcoholic fatty liver disease, and hepatic cirrhosis. Patients with focal nodular hyperplasia, hepatic cysts, hepatic adenoma, hemangioma, or hepatocellular carcinoma were included in the test dataset-1. Patients with large hepatic masses (including cholangiocarcinoma, hemangioma, hepatocellular carcinoma, etc.) who were classified as candidates for major hepatectomy were included in dataset 2. The characteristics of the three datasets are shown in Table 1.

**Table 1:** Characteristic of training and test datasets

Parameters	Training Dataset	Test Dataset-1	Test Dataset-2
No. of patients	170	146	32
No. of male patients (*n*, %)	106 (62.35)	84 (57.53)	25 (78.13)
Age (year)	50.23 ± 13.77	49.04 ± 13.15	54.59 ± 14.29
Average Volume of intrahepatic lesions (cm^3^)	2.02 ± 4.13 (0.00–49.40)	1.87 ± 7.92 (0.00–86.05)	448.80 ± 608.60 (9.14–2426.10)
Liver conditions (*n*, %)			
Reported healthy liver	61 (35.88)	50 (34.25)	27 (84.38)
Fatty liver	62 (36.47)	47 (32.19)	3 (9.38)
Hepatic cirrhosis	47 (27.65)	49 (33.56)	2 (6.25)

### Imaging acquisition

CT images were obtained by five CT scanners from three different manufacturers (summarized in Appendix E1 (supplement)). CT images reconstructed at section thicknesses of 1.25 mm and 1 mm were included in this study.

### Imaging processing and labelling

**Fig. 2:**
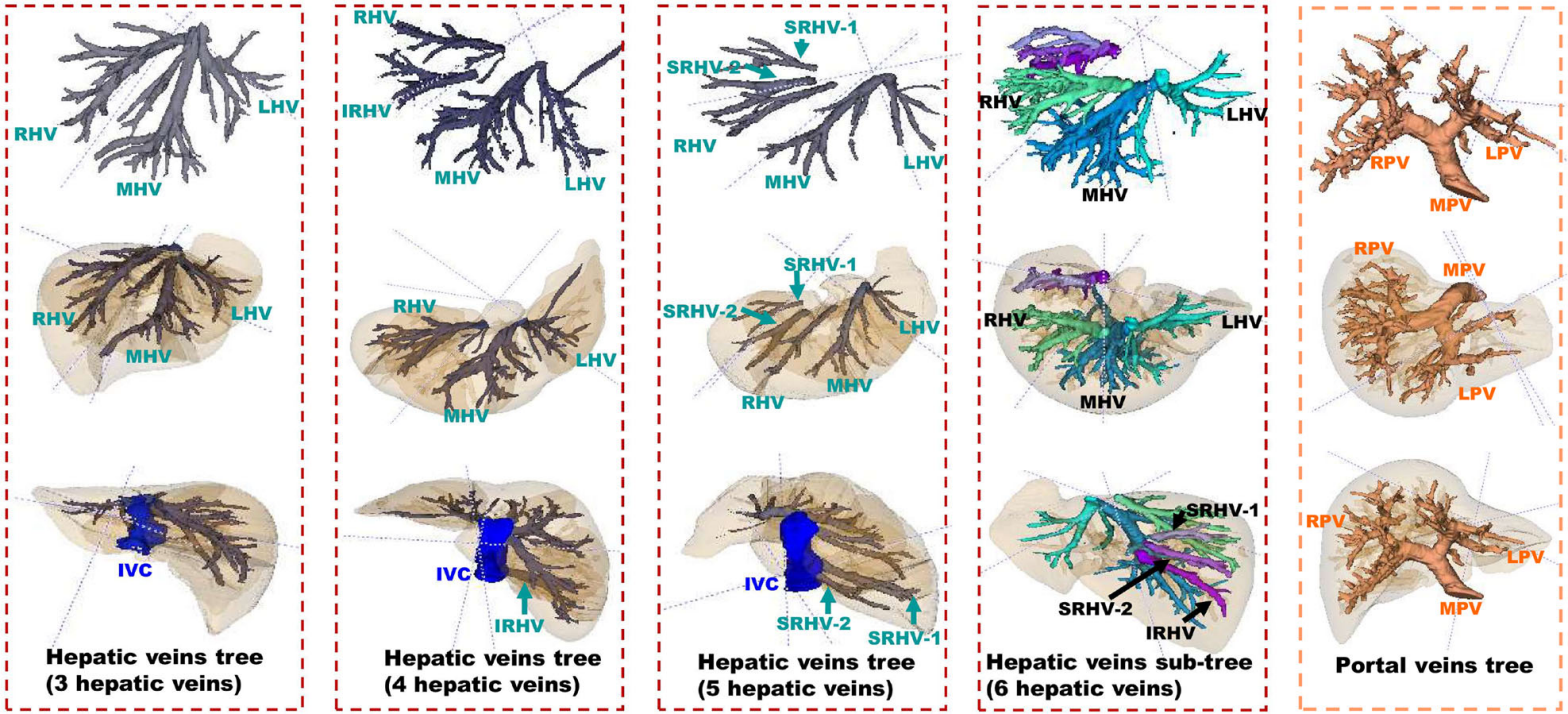
Major hepatic veins including right hepatic vein (RHV), middle hepatic vein (MHV), left hepatic vein (LHV), superior RHV (SRHV) and inferior RHV (IRHV)) were annotated. Main portal vein (MPV), Left portal vein (LPV)) and right portal vein (RPV) were annotated. Inferior vena cava (IVC) was labelled as a reference landmark of the hepatic veins

We used ITK Snap version 3.8.0 for imaging processing. Major hepatic veins (i.e., the right hepatic vein (RHV), middle hepatic vein, left hepatic vein, superior RHV and inferior RHV were annotated up to the second branch ramification. The main portal vein was fully annotated. The left portal vein and right portal vein were annotated up to the second branch of the ramification (shown in Fig. 2).

All the labels were first annotated by a radiologist and subsequently re-evaluated and corrected by another radiologist (with 10 years of experience in liver imaging and 30 years of experience in radiology); these annotations were regarded as the ground truth. All the images from the training and external validation datasets were processed via this procedure.

DL models for the automated segmentation of the entire liver, Couinaud’s liver segments and hepatic mass were trained in our institution for a precise preoperative assessment of FLR% _*B-free*_, the dataset cohorts and performances are summarized in Appendix E1 (supplement).

**Fig. 3:**
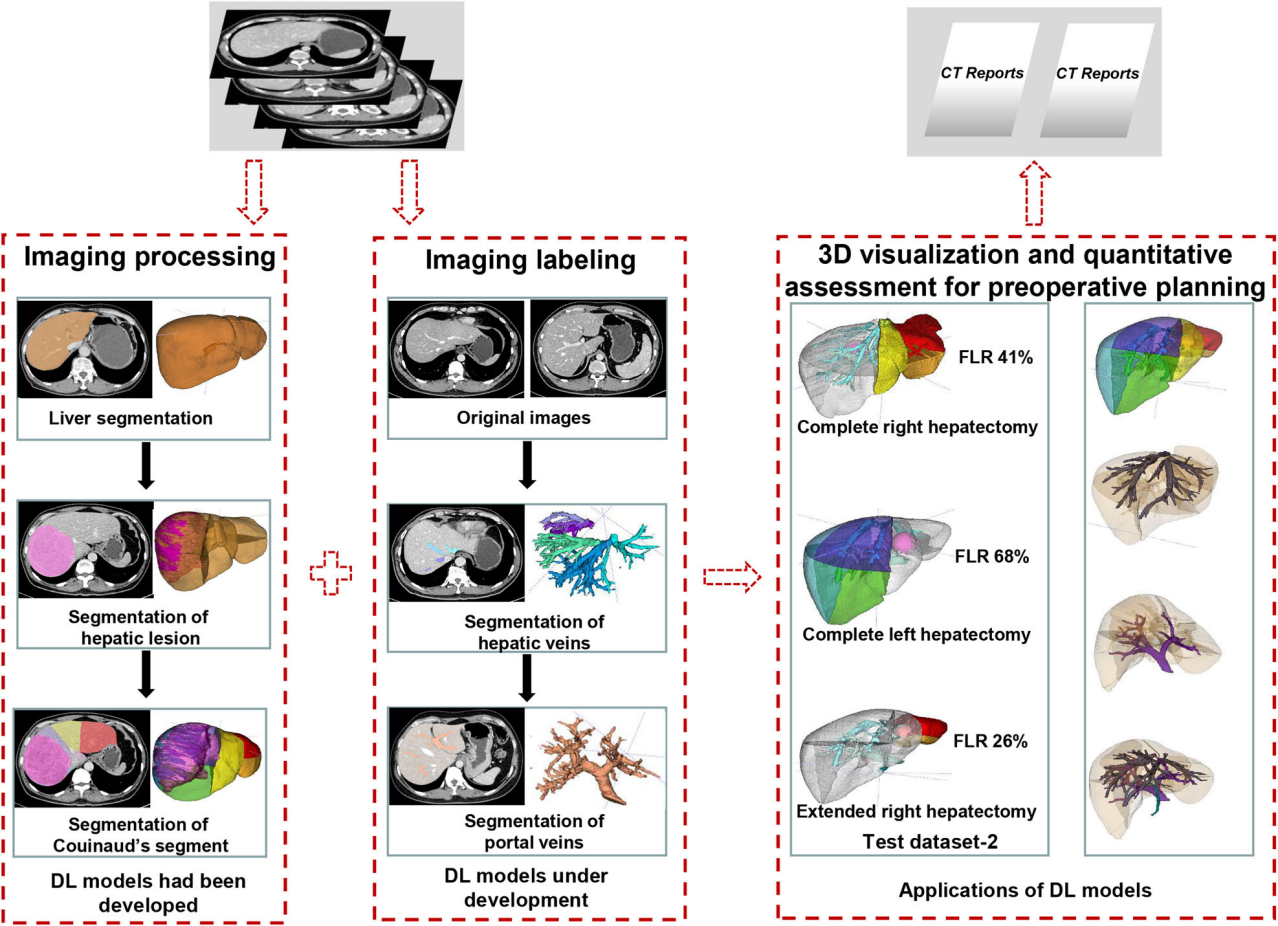
Key steps in 3D visualization and quantitative future liver remnant (FLR) assessment for preoperative planning

Three-dimensional visualization and quantitative assessment of FLRs were proposed. The key steps are demonstrated in Fig. 3.

### Model development

The 3D U-Net network described by Çiçek Ö et al [19] was used for the development of DL models of hepatic veins and portal veins. Another three 3D U-Net frameworks were trained for the segmentation of the entire liver, Couinaud’s segments, and hepatic mass (Appendix E1 (supplement)).

For the development of 3D U-Net models for hepatic and portal veins, 3D contrast-enhanced CT images were inputted with manual annotation of all veins and branches with a diameter larger than 2 mm, and the output was produced with the predicted annotation. Training, validation, and test data were combined at an 8:1:1 ratio for the development dataset. We use the Dice loss function as the loss function. The prediction accuracy of the DL models was checked on the validation dataset during the training process. We stopped our training when the prediction accuracy started to decrease to prevent overfitting. The resolution of the CT images was 128 × 192 × 256. Image amplification methods, including translation, affine transformation, and random noise were adopted. During the model training, the ADAM gradient descent optimization algorithm was adopted, with a batch size of 2, an initial learning rate of 0.0001 and 400 epochs. We used Python as the programming language. The software used was PyTorch 0.4.1, Python 3.6, Numpy, OpenCV and SimpleITK, and the hardware used was an NVIDIA Tesla P100 16 G GPU for model training.

### Model evaluation and qualitative assessment

- *For classification of trunks and branches of hepatic and portal veins.* The automated results were regarded as accurate when 3/4 of the length of the main portal veins and hepatic veins and 1/2 of the length of the primary branches were accurately and continuously annotated.
- *For segmentation of hepatic and portal veins*, we used Dice similarity coefficient (DSC) and volumetric similarity (VS) to evaluate the segmentation performance. The DSC was calculated as the voxel overlap between the ground truth (*G*) and the prediction masks (*p*).
  $${DSC}=\frac{2{{{{{\rm{|}}}}}}P\cap G{{{{{\rm{|}}}}}}}{{{{{{\rm{|}}}}}}P{{{{{\rm{|}}}}}}+{{{{{\rm{|}}}}}}G{{{{{\rm{|}}}}}}}$$
  The VS is used to measure the volumetric difference between the ground truth and prediction masks (i.e., *V*_*G*_ and *V*_*p*_).
  $${VS}=1-\frac{|{V}_{G}-{V}_{P}|}{|{V}_{G}+{V}_{P}|}$$

c. *Calculation of the FLR%* The total liver volume (*TLV*), *FLR* and hepatic lesion (*V*_*Lesion*_) were measured on CT images. The ratio of *FLR* to the nontumor-bearing liver volume was defined as *FLR%* [20].
  $${FLR} \% =\frac{{FLR}}{{TLV}-{V}_{{Lesion}}}\times 100 \%$$
  $${{FLR} \% }_{\,\, B-{free}}=\frac{{{FLR}}_{B-{free}}}{{{TLV}}_{B-{free}}-{V}_{{Lesion}}}\times 100 \%$$
  $${{FLR} \% }_{\,\, B-{filled}}=\frac{{{FLR}}_{B-{filed}}}{{{TLV}}_{B-{filled}}-{V}_{{Lesion}}}\times 100\, \%$$
  FLRs were calculated in the settings of blood-free (FLR% _*B-free*_) and blood-filled (FLR% _*B-filled*_) for each patient in test dataset-2.

d. *The prediction of resection based on the FLR%*_*B-free*_ *status and FLR%*_*B-filled*_ *status* The optimal minimal FLR% varies for different liver pathologies, and FLR% values larger than 20%, 30%, and 40% in patients with healthy livers, hepatic steatosis, and cirrhosis, respectively, were classified as candidates for major hepatectomy in this study [21–23].

### Statistical analysis

For the DL models of hepatic and portal veins, to evaluate the accuracy of trunk and branch classification, the automated and manually labelled classification results were compared. To assess the accuracy of the segmentation and volumetry, the DSC and VS values between the automated and manual segmentation methods were compared. To evaluate the ability of the DL models to assess the preoperative FLR, the differences in FLR% between automated and manual segmentation and between blood-filled and blood-free settings were compared via Bland–Altman analysis. The differences in the prediction of resection between the model and human doctors and between blood-filled and blood-free settings were compared using McNemar’s test. Commercially available software (GraphPad Prism, version 7.00; IBM SPSS Statistics for Mac, version 22.0) was used to perform the statistical analysis, and a *p* value less than 0.05 was considered to indicate statistical significance.

## Results

### Classification accuracy of trunks and branches of hepatic and portal veins

The results of test datasets 1 + 2 show that our model is capable of accurately classifying the input contrast-enhanced liver CT images into hepatic veins and portal veins, including trunks and branches. The classification accuracy for the trunks and branches ranged from 81.46% (145/178) to 98.88% (176/178) (shown in Table 2). Unsatisfactory classifications and the accuracy of accessory right hepatic veins were shown in Appendix E2 (supplement).

**Table 2:** Accuracy of deep learning model in the classification of trunk and branches of hepatic and portal veins in test dataset 1 + 2 (%, 95% confidence intervals)

	RHV	MHV	LHV	SRHV	IRHV	MPV	RPV	LPV
Trunk	98.88 (97.31, 100.00)	96.63 (93.95, 99.31)	97.75 (95.55, 99.95)	96.07 (93.18, 98.95)	95.51 (92.43, 98.58)	88.20 (83.42, 92.99)	88.20 (83.42, 92.99)	87.64 (82.76, 92.52)
Branches	98.31 (96.41, 100.00)	96.07 (93.18, 98.95)	97.19 (94.74, 99.64)	94.38 (90.97, 97.80)	94.94 (91.69, 98.19)	NA	82.58 (76.96, 88.21)	81.46 (75.70, 87.23)

*RHV* right hepatic vein, *MHV* middle hepatic vein, *LHV* left hepatic vein, *SRHV* superior right hepatic vein, *IRHV* inferior right hepatic vein, *MPV* main portal vein, *LPV* left portal vein, *RPV* right portal vein

#### The accuracy of the segmentation of hepatic and portal veins in test dataset 1 + 2

The average DSCs for the segmentation of hepatic veins and portal veins were 0.66 ± 0.08 (95% CI: (0.65, 0.68)) and 0.67 ± 0.07 (95% CI: (0.66, 0.69)), respectively, and the average VS was 0.80 ± 0.10 (95% CI: (0.79, 0.84)) and 0.74 ± 0.08 (95% CI: (0.73, 0.76)), respectively.

**Fig. 4:**
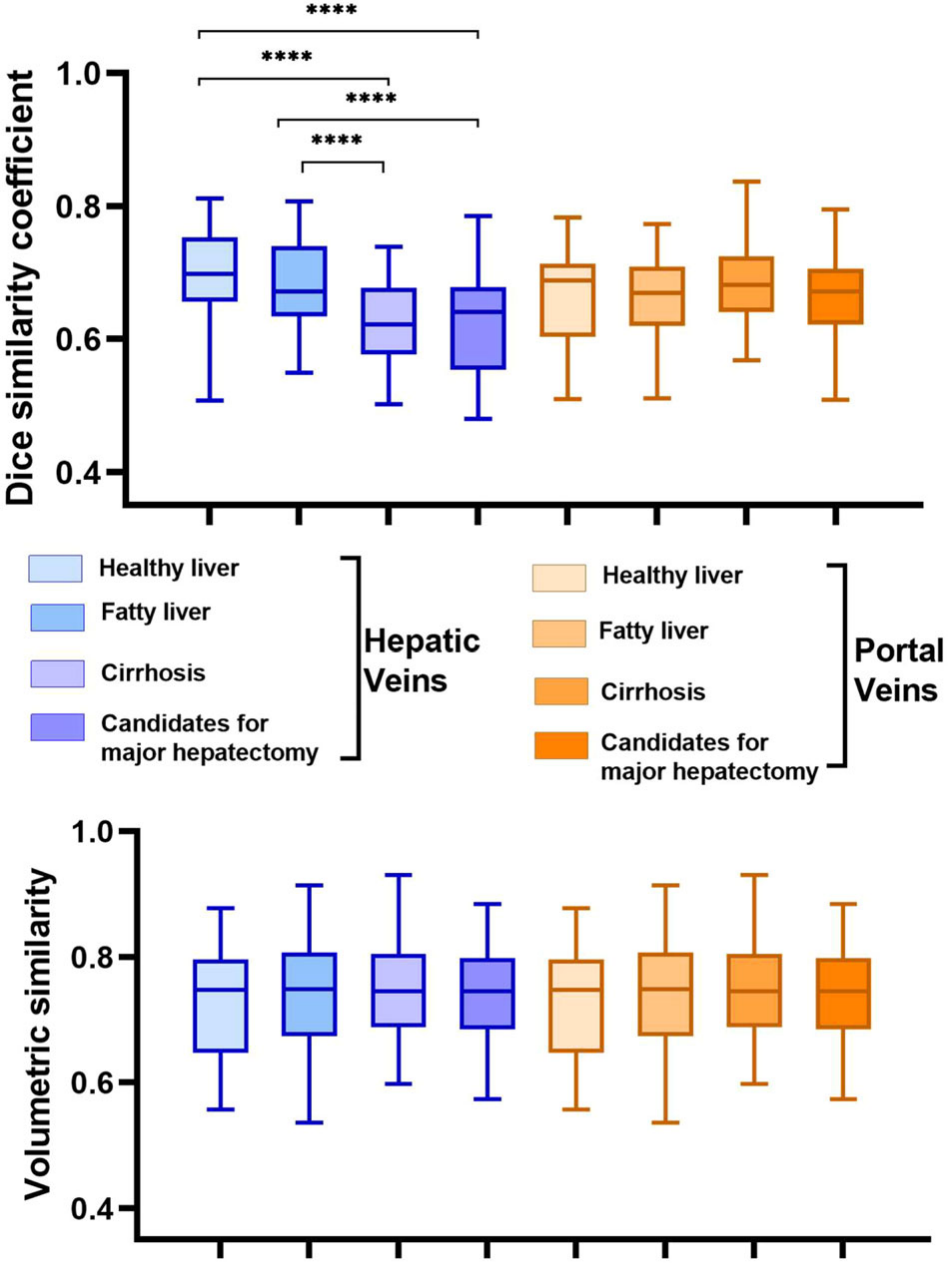
Box and whisker plot shows the medians of DSC values ranged from 0.62 to 0.70 in subgroups of healthy liver, fatty liver, hepatic cirrhosis and candidates for major hepatectomy in the segmentation of hepatic veins, respectively. For the results of DSC, the differences between subgroups were statistically significant (all *p* < 0.0001), but no significant differences in the segmentation of portal veins segmentation were found among groups (*p* = 0.689). For the results of VS, the median values ranged from 0.75 to 0.86, no significant differences among subgroups in both segmenting hepatic veins and portal veins were found (*p* = 0.749 for hepatic veins, *p* = 0.932 for portal veins, respectively)

According to the DSC results, the differences in the segmentation of hepatic veins between healthy livers and cirrhotic livers, healthy livers and candidates for major hepatectomy, fatty livers and cirrhosis, and fatty livers and candidates for major hepatectomy were statistically significant (*p* < 0.0001), but no significant differences in portal vein segmentation were found among the subgroups (*p* = 0.689). For the VS results, no significant differences in segmenting hepatic veins or portal veins were found among the subgroups (*p* = 0.749 for hepatic veins, *p* = 0.932 for portal veins) (Fig. 4).

For segmentation performance, our results were compared with those of similar studies (Table 3). We validated our models by using the largest test dataset, which included data from most liver conditions. For segmentation of hepatic veins, we obtained similar results to those of Tong [15], Tong [17] and Oh [24], with differences of less than 0.3 in DSC. For the segmentation of portal veins, we obtained the highest DSC values.

**Table 3:** Segmentation performance of hepatic vein (HV) and portal vein (PV) compared with literature

First Author/ Year	Modality	Segmentation Methodology	Test Dataset Size	Liver Diseases	DSC in test Dataset (HV/PV)	Vessels
Tong/2023 < 15 >	CT	CM U-Net	80	NA	0.70/NA	Only HV
Tong/2023 < 17 >	CT	SDA U-Net	80	Liver tumors and calcification	0.71/NA	Only HV
Zbinden/2022 < 28 >	MR	3D nnU-Net	30	With/without chronic liver disease	0.532 /0.634	HV/PV
Oh/2023 < 24 >	MR	3D Residual U-Net	12	Liver tumors	0.70/0.61	HV/PV
Proposed	CT	3D U-Net	178	Healthy livers, hepatic steatosis, cirrhosis and candidates for major hepatectomy	0.67/0.68	HV/PV

#### Volumetric accuracy of large blood vessels in test dataset 1 + 2

**Fig. 5:**
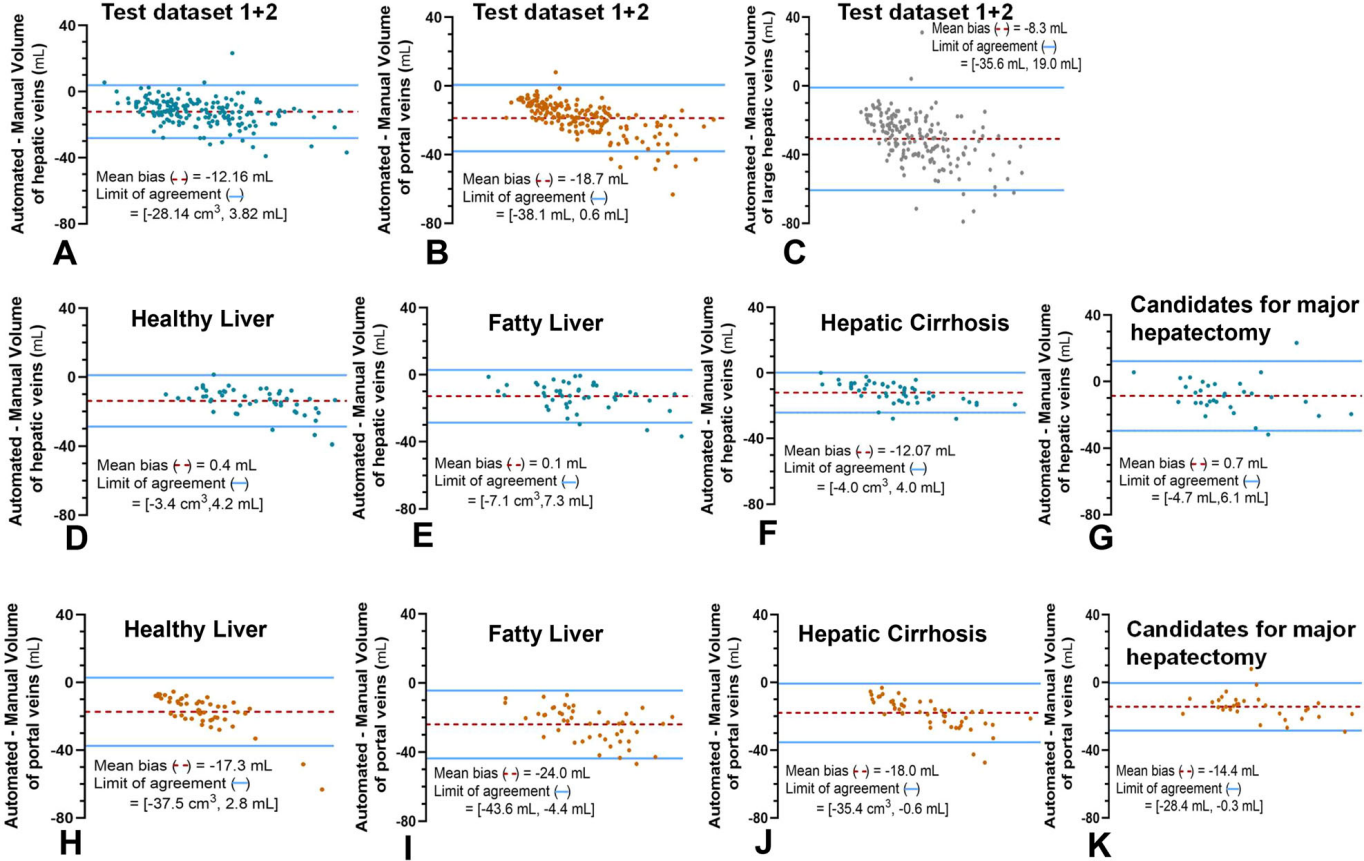
Bland-Altman plots for agreement between manual and automated method in volumetry of hepatic veins (**A**, **D**, **E**, **F**, **G**), portal veins (**B**, **H**, **I**, **J**, **K**) and large hepatic veins (**C**). The segmentation models slightly underestimated manual segmentations in healthy liver, fatty liver, hepatic cirrhosis and candidates for major hepatectomy

The average volumes of the hepatic veins and portal veins obtained by automated and manual segmentation are shown in Table 4. Volumetry of hepatic veins obtained by automated methods underestimated manual results in all patients (bias was −12.93 mL and −8.67 mL, *p* < 0.05; 95% limits of agreement (LoA) were −27.25 mL and 1.40 mL; and −29.62 mL and 12.29 mL in test dataset-1 and test dataset-2, respectively). Volumetry of the portal veins obtained by automated methods underestimated the manual results in all patients (bias was −19.69 mL and −14.36 mL, *p* < 0.05; 95% LoA were −39.53 mL and 0.15 mL; and −28.42 mL and −0.30 mL in test dataset 1 and test dataset 2, respectively) (Fig. 5).

**Table 4:** Volumetry of hepatic veins and portal veins in the Test dataset 1 + 2. (Mean standard deviation (95% confidence interval))

	Hepatic Veins					Portal Veins				
	Test Dataset-1			Test Dataset-2	Test Dataset 1 + 2	Test Dataset-1			Test Dataset-2	Test Dataset 1 + 2
	Healthy Liver	Fatty liver	Cirrhosis			Healthy Liver	Fatty liver	Cirrhosis		
Average Volume (M) (mL)	38.99 (35.84, 42.15)	47.53 (43.38, 51.68)	26.68 (24.01, 29.35)	28.78 (24.48, 33.08)	36.02 (33.90, 38.14)	38.01 (34.31, 41.71)	58.68 (54.32, 63.04)	41.66 (38.10, 45.22)	36.06 (32.31, 39.81)	44.12 (41.81, 46.44)
Average Volume (A) (mL)	25.17 (23.14, 27.21)	34.66 (31.48, 37.84)	14.61 (12.97, 16.25)	20.11 (16.63, 23.60)	23.86 (22.19, 25.53)	20.71 (19.20, 22.21)	34.67 (31.78, 37.57)	23.67 (21.89, 25.45)	21.70 (18.99, 24.41)	25.39 (24.02, 26.76)

Average Volume (M): Average volume obtained by manual segmentation; Average Volume (A): Average volume obtained by automated segmentation

#### Volumetric accuracy of FLR and FLR% in test dataset 2

**Fig. 6:**
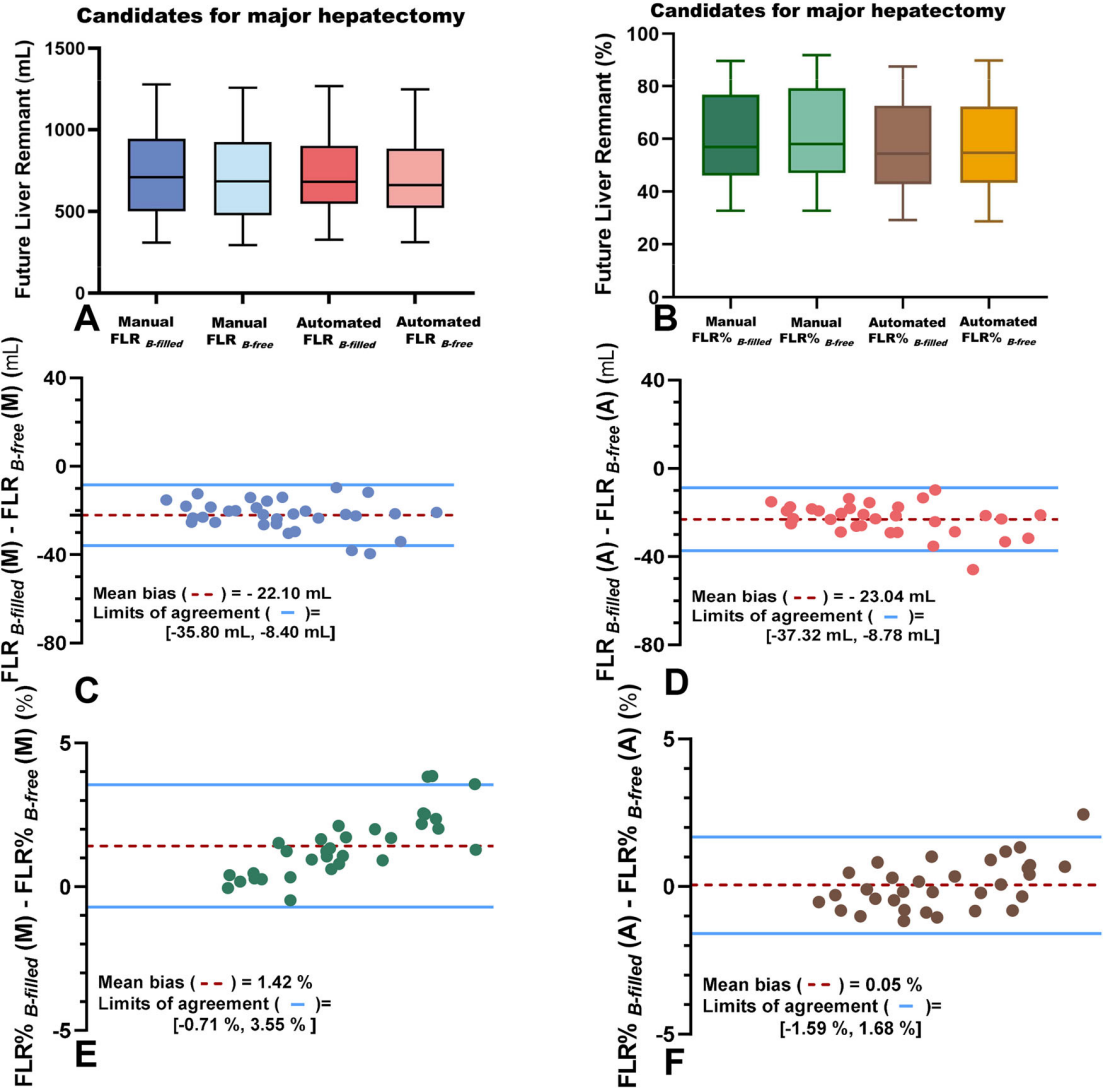
Box and whisker plot shows preoperative *FLR*_*B-free*_ and *FLR*_*B-filled*_ (**A**), *FLR*%_*B-free*_ and *FLR%*_*B-filled*_ (**B**) in candidates for major hepatectomy obtained by using manual and automated methods. The central boxes, the middle lines in the central boxes represent the values from 25th to 75th percentile, the medians, respectively. Vertical lines under and upper the boxes extended from the minimum values to the maximum values. Bland-Altman plots for agreement between FLR _*B-free*_ and FLR _*B-filled*_ by using manual (**C**) and automated (**D**) method; Bland-Altman plots for agreement between FLR% _*B-free*_ and FLR% _*B-free*_ by using manual (**E**) and automated (**F**)

The FLR and FLR% assessments are shown in Fig. 6, respectively. No significant differences FLR in _*B-free*_ or FLR _*B-filled*_ values were noted between the manual and automated methods using the Mann–Whitney *U* test (*p* = 0.67 and 0.66, respectively) (Fig. 6). No significant differences FLR % in *B-free* or FLR % _*B-filled*_ were noted between the manual and automated methods using the Mann–Whitney *U* test (*p* = 0.59 and 0.99, respectively).

In the blood-filled setting, the volumetry of the FLR _*B-filled*_ and FLR% _*B-filled*_ samples ranged from 309.87 to 1277.00 mL (mean volume, 725.99 mL ± 253.09) and 32.82% to 89.67% (mean value, 59.19% ± 16.56%), respectively. In the blood-free setting, the FLR _*B-free*_ and FLR% _*B-free*_ volumes ranged from 294.63 to 1256.10 mL (mean volume, 703.89 mL ± 251.06) and from 32.77% to 91.87% (mean value, 60.61% ± 17.43%), respectively (Fig. 6).

FLR assessments obtained by the blood-free setting slightly underestimated the FLR _*B-filled*_ by using manual and automated methods (bias = −22.1 mL, −23.04 mL, *p* < 0.01; 95% LoA = −35.80 mL and −8.40 mL; −37.32 mL and −8.78 mL, respectively); FLR assessments obtained by the blood-free setting slightly overestimated the FLR% _*B-filled*_ by using manual and automated methods (bias = 1.42%, 0.05%, *p* < 0.05; 95% LoA were −0.71% and 3.55%, −1.59% and 1.68%, respectively) (Fig. 6).

For the volumetric volume of the FLR, we compared our results with those of similar studies via volumetry of the right lobe (shown in Appendix E2 (supplement)). We obtained similar results with Kim’s [8] and Kalshabay’s [10] methods, with differences of less than 45 mL (accounting for 5.59% of the TLV). However, our results were quite different from those of Gündoğdu’s [25] and Park’s [9] studies, in which the difference was more than 450 mL (accounting for 55.90% of the TLV). The differences in the volumes of the right lobe may be related to the differences in the study population and the differences in the calculation methods used for the FLR in these studies. Patients enrolled in Gündoğdu’s [25] and Park’s [9] studies were living liver donors with no hepatic disease or mild fatty liver disease; these findings are quite different from our study population.

## Qualitative analysis results

### Comparison of the prediction of resection in test dataset 2

A total of 10 patients, 21 patients and 1 patient underwent complete left hepatectomy, complete right hepatectomy and extended right hepatectomy, respectively. A total of 128 (32 × 2 × 2) FLR% measurements were obtained and compared. The number of patients categorized as candidates for resection is shown in Table 5. All patients were permitted to undergo major hepatectomy via manual or automated segmentation, based on FLR% _*B-free*_ or FLR% _*B-filled*_ assessment results. No significant differences in the prediction of resection were found between the human doctors and the automatic segmentation model (*p* > 0.99) or between the FLR% _*B-free*_ assessment and the FLR% _*B-filled*_ assessment (*p* > 0.99) according to McNemar’s test.

**Table 5:** Number of cases categorized as candidates for major hepatectomy

Methods	Based on FLR% _*B-free*_	Based on FLR% _*B-filled*_
Manual segmentation	32	32
Automated segmentation	32	32

FLR% _*B-free:*_ the ratio of future liver remnant to total liver volume measured in blood free setting; FLR% _*B-filled:*_ the ratio of future liver remnant to total liver volume measured in blood filled setting

## Discussion

Preoperative CT volumetry of FLR has been criticized for under- and over-estimating real FLR mainly because volumetry on CT images is blood-filled while intraoperative volumetry is blood-free [4, 6, 7]. The larger the volume of the blood vessels, the greater the difference between measured FLR and real FLR will be. Blood vessels account for 9% of total liver volume [8], a proportion that has the potential to change the prediction of resection based on FLR% because the minimum FLR% which required to preserved ranged from 20% to 40% before major hepatectomy. So, a precise FLR calculation in a blood-free setting, and a comparison between FLR _*B-filled*_ and FLR _*B-free*_ are essential. In this study, we developed and validated DL models for the automatic segmentation of hepatic veins and portal veins and applied this technique for presurgical FLR% assessment both in a blood-filled setting and a blood-free setting prior to major hepatectomy. The key contributions of this study were that preoperative FLR% assessments and predictions of resection in blood-filled and blood-free settings were fully compared with quantitative and qualitative results, multiple types of hepatectomy were included, and the validation dataset included patients with various liver conditions in clinical practice and candidates who underwent major hepatectomy.

The current results indicated that our model allowed fully automatic segmentation of hepatic veins and portal veins and fully automatic volumetry of FLR% in a blood-free setting and was robust for different pathological livers, even in a spatial external validation dataset.

Our models obtained slightly higher DSC values than Zbinden et al [26] and Oh et al [24] (Table 3). For the segmentation of hepatic veins, our models obtained DSC values similar to those of Tong et al [15] and Tong et al [17]. We obtained higher DSC values in patients with healthy livers and fatty livers than in patients with hepatic cirrhosis and large liver tumors (shown in Fig. 4). This was primarily because the hepatic veins in patients with cirrhosis and liver tumors were smaller, more blurred and more difficult to distinguish than those in patients with healthy livers, which increased the difficulty in segmenting hepatic veins.

For the calculations of both the FLR and FLR%, similar studies have focused on the correlations between FLR weight and remnant liver weight [8, 27]; however, the differences between FLR _*B-filled*_ and FLR _*B-free*_ weight have rarely been analyzed. Our study demonstrated that automated preoperative assessment of FLRs that are B-free and FLR % _*B-free*_ is feasible, and both results could be used in the prediction of major hepatectomy.

Limitations in our study should be noted. First, for the external validation of the preoperative FLR _*B-free*_ assessment, the validation would be stronger if the volume of the actual liver remnant after hepatectomy was obtained and regarded as a reference. Second, validation on unseen pathologies was lacking, and the FLR was validated in 32 patients who underwent three types of major hepatectomy. Further validation in a larger dataset involving unseen pathologies more types of major hepatectomy is needed. In conclusion, fully automated preoperative assessments of FLRs in blood-free settings are feasible prior to major hepatectomy, even for different types of resection and various liver conditions. Compared to those of human doctors, the DL models demonstrated similar performance in the final prediction of resection in a spatial external validation dataset.

### Supplementary information

ELECTRONIC SUPPLEMENTARY MATERIAL

## Data Availability

The datasets used and/or analyzed during the current study are available from the corresponding author (E-mail: Benliyong@126.com) on reasonable request.

## Abbreviations

3D: 3-dimensional
95% LoA: 95% limits of agreement
DL: Deep learning
DSC: Dice similarity coefficient
FLR _B-filled_: Future liver remnant in a blood-filled setting
FLR _B-free_: Future liver remnant in a blood-free setting
FLR: Future liver remnant
G: Ground truth
IRHV: Inferior right hepatic vein
P: Prediction masks
PHLF: Posthepatectomy liver failure
RHV: Right hepatic vein
SRHV: Superior right hepatic vein
TLV: Total liver volume
V blood: Volume of portal veins and hepatic veins
V_G_: Volume of the ground truth
V_Lesion_: Volume of hepatic lesion
V_P_: Volume of the prediction masks
VS: Volumetric similarity
