# Supplementary material for: Fully automated assessment of the future liver remnant in a blood-free setting via CT before major hepatectomy via deep learning
Source: Insights Imaging. 2024 Jun 27;15:164. doi: 10.1186/s13244-024-01724-6 (PMC11211293; doi:10.1186/s13244-024-01724-6)
Supplement: Supplementary file 1 — ELECTRONIC SUPPLEMENTARY MATERIAL [file 13244_2024_1724_MOESM1_ESM.pdf]

Fully automated assessment of the future liver remnant in a blood-free setting via CT before major hepatectomy via deep learning

ELECTRONIC SUPPLEMENTARY MATERIAL

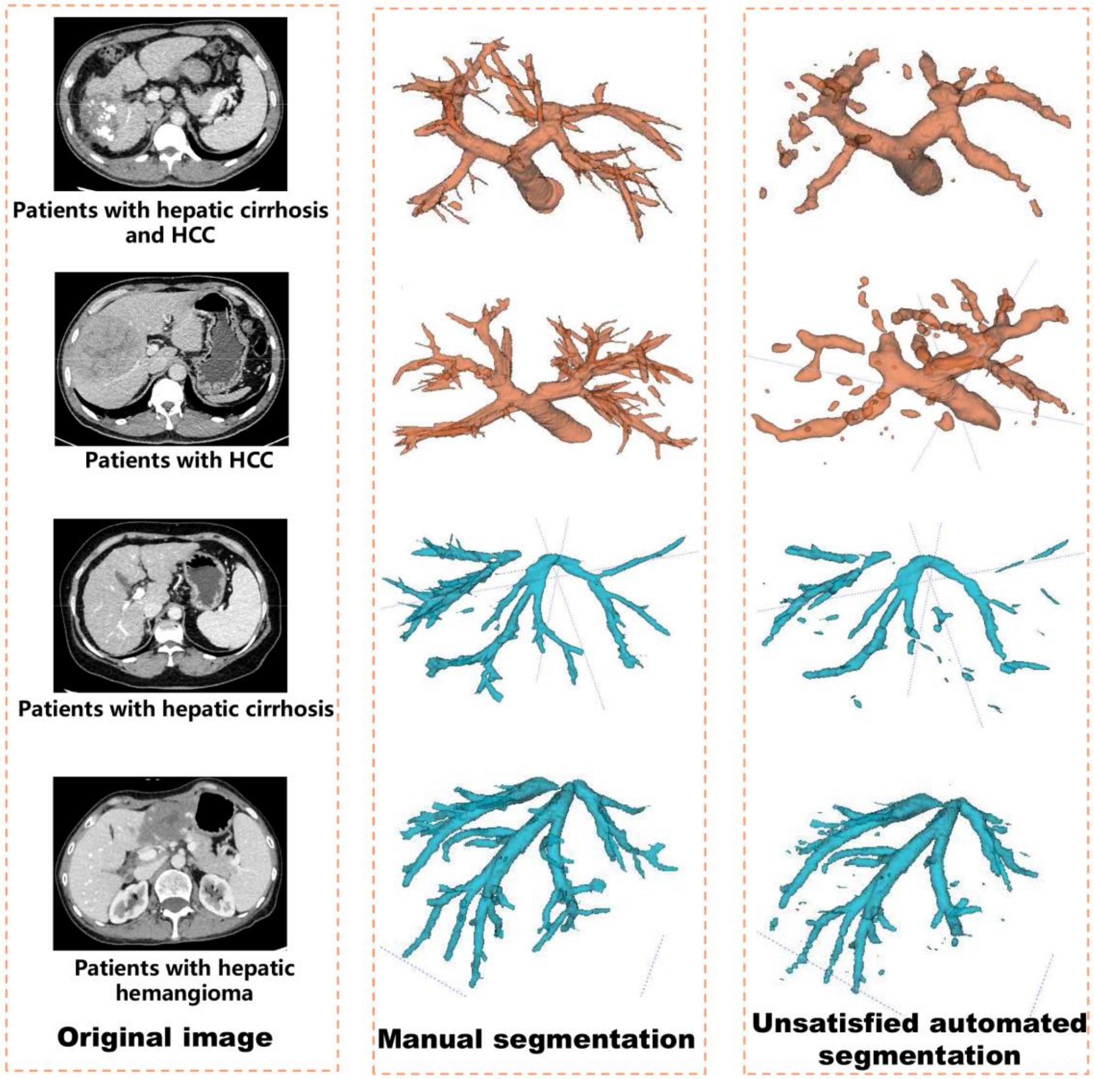

Figure 1. Unsatisfied automated segmentation results of hepatic veins and portal veins in cases with hepatic cirrhosis and in candidates for major hepatectomy. Unsatisfactory classifications mostly occurred in patients with hepatic cirrhosis and large hepatic masses because of misidentification and inconsecutive automated annotations.

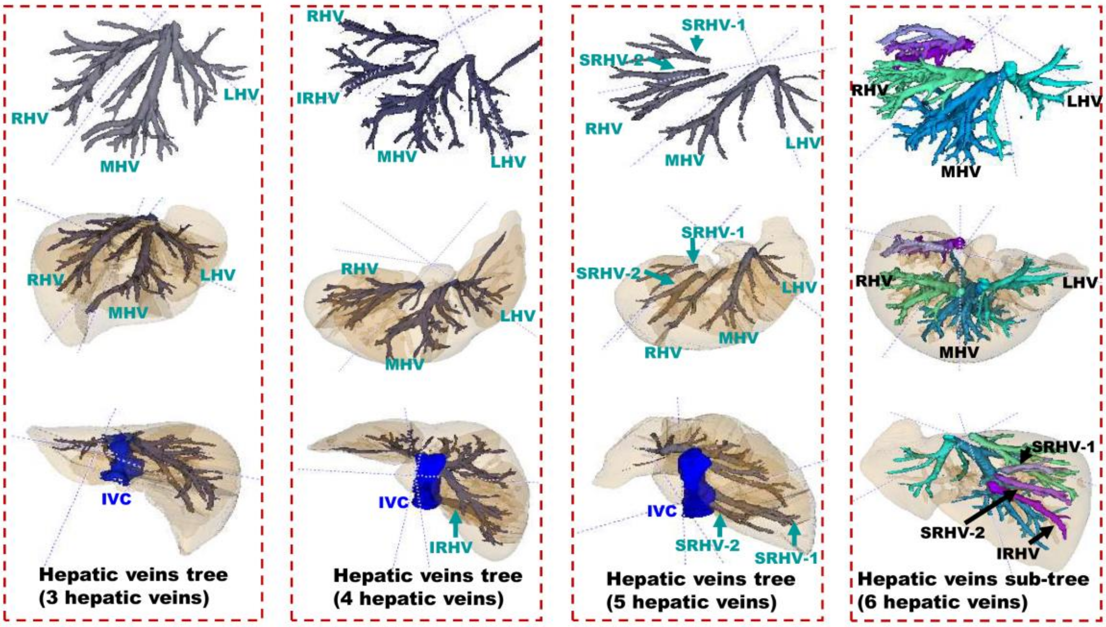

Figure 2. Classification of accessory right hepatic veins.

There were 12 cases presented with one inferior right hepatic vein (IRHV), 12 cases presented with one IRHV and two superior right hepatic veins (SRHV), 3 cases presented with 2 SRHVs. Other cases have three hepatic veins, named the right hepatic vein (RHV), middle hepatic vein (MHV) and left hepatic vein (LHV). The marking of the inferior vena cava (IVC) is used to assist in the identification of the origin of the hepatic vein.

There were 24 IRHVs presented in 24 cases. There were 154 cases absence of IRHV. Our model predicted 16 IRHVs and 154 cases absence of IRHV accurately, with the accuracy of 95.51% [(16+154)/178×100%]. The predicted result of 8 IRHVs were unsatisfied and were regarded as inaccurate.

There were 30 SRHVs presented in 15 cases. There were 12 cases presented with 1 IRHV and 2 SRHVs, and 3 cases presented with 2 SRHVs. There were 163 cases absence of SRHV. Our model predicted 22 SRHVs (in 11 cases) and 160 cases absence of SRHV accurately, with the accuracy of 96.07% [(11+160)/178×100%]. The predicted result of 8 IRHVs (in 4 cases) were unsatisfied and were regarded as inaccurate.

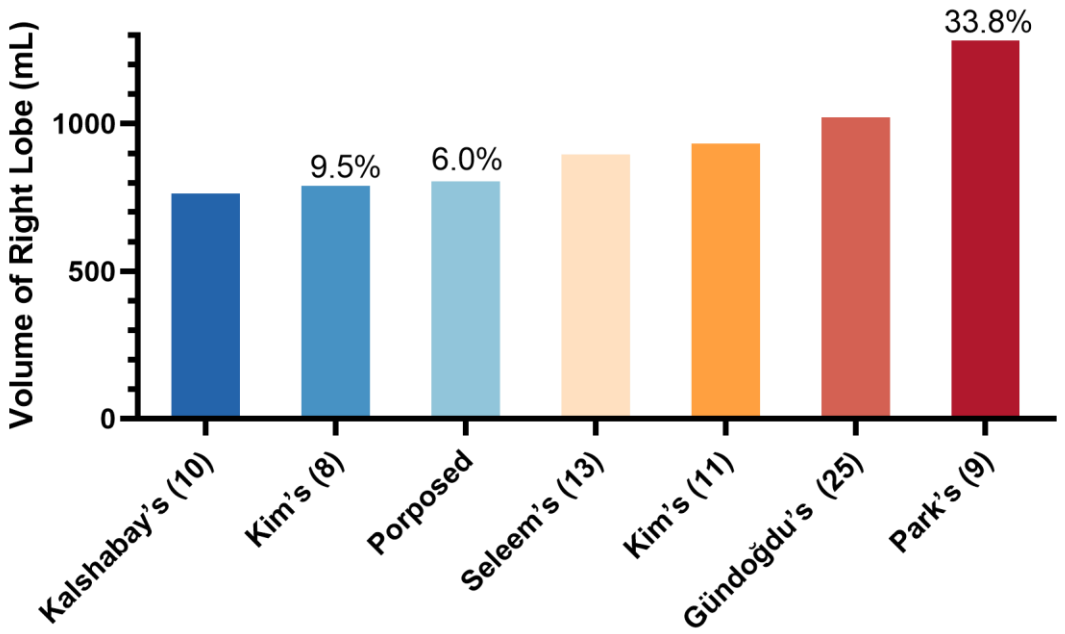

Figure 3. Volumetry of right lobe was compared with similar studies. We obtained similar results with Kim's (8) and Kalshabay's (10) with a difference of less than 45 mL, but quite different from Gündoğdu's (25) and Park's (9). The volume of large blood vessels accounted for 6.0%, 9.5% and 33.8% of TLV in our, Kim's and Park's study.

Table 1: Characteristics of the CT scans.

Parameters	Training Dataset (n=170)	Test Dataset-1 (n=146)	Test Dataset-2 (n=32)
CT Imaging system			
Siemens Definition Flash	25	53	18
Philips Briliance iCT 256	72	NA	NA
GE Light Speed VCT	39	NA	NA
GE Discovery CT750 HD	34	NA	NA
GE Revolution	NA	93	14
Imaging thickness (mm)	1.0	1.0/1.25	1.0/1.25
Tube voltage			
120 kVp	114	108	27
100 kVp	56	38	5
Images			
Portovenous phase	170	135	24
Delay phase	0	11	8

Table 2: Performance of DL models in the segmentation of liver, hepatic mass and Couinaud's segment in test dataset 1+2.

Parameters	Liver segmentation	Hepatic Lesion segmentation	Segmentation for Couina'd's segment
Cohort (CT scans/Patients)			
Train	2375/307	492/492	170/170
External validation	178/178	32/32	178/178
Liver conditions (CT scans: Train/Validation)			
Reported healthy liver	1011/50	207/27	61/50
Fatty liver	592/47	142/3	62/47
Hepatic cirrhosis	307/49	143/2	47/49
Candidates for major hepatectomy	0/32	0/32	0/32
Intrahepatic bile duct dilatation	205/2	15/2	5/2
Post-hepatectomy	260/0	0/0	0/0
Performance			
DSC	0.984 ± 0.011	0.693 ± 0.247	0.94 ± 0.00
Volumetric similarity	0.998 ± 0.010	0.784 ± 0.213	NA

Development Dataset

A total of 1942 CT scans in 1942 patients in Medical Imaging Center of Peking University First Hospital were included in the training dataset. CT data from healthy livers, hepatic steatosis and cirrhosis was included in order to develop a robust deep learning model that performs well in various liver conditions in clinical practice. It was obtained in multiple steps from a large cohort of patients with CT data from a previous study (1).

First, we continuously include the first 61 subjects with a normal liver (i.e., no clinical and pathologic evidence of liver disease) who accepted CT scan between January 2018 and March 2019. For 62 subjects with fatty liver, we continuously included the first 20 subjects of mild fatty liver (i.e., the MRI proton density fat fraction (PDFF) measurement results ranged from 6.5% to 17.4%, and the time interval between MR PDFF examination and CT examination is less than 2 weeks), the first 20 subjects of moderate fatty liver (i.e., MRI PDFF measurement results ranged from 17.5% to 22.1%), and the first 22 subjects of severe fatty liver (i.e., MRI PDFF measurement results higher than 22.1%) who accepted CT scan between January 2018 and March 2019 (2). For 47 subjects with hepatic cirrhosis, we continuously included the first 47 subjects with Child-Pugh Classification of A cirrhosis. Because patients with Child-Pugh Classification of B and C were not recommended to accept hepatectomy (3).

Data description, preprocessing and deep learning algorithm

We develop 3D U-Net network described by Çiçek Ö et al. (4) for the segmentation of hepatic veins and portal veins. For the development of 3D U-Net models for hepatic and portal veins, 3D contrast-enhanced CT images were inputted with manual annotation of all veins and branches with a diameter larger than 2 mm, and the output was produced with the predicted annotation.

We extract slices with blood vessel annotations because the original image is a liver CT image, but the annotations for blood vessels are concentrated in slices with liver tissue. In order to reduce the requirement of GPU memory size during training, the resolution of the CT images was set to 128×192×256, which ensures the integrity of the tiny branches of hepatic veins and portal veins. By counting the CT values corresponding to the existing tags in the dataset, we set the window width of the CT image to 300 and the window level to 30. Selecting the threshold range can effectively reduce the erroneous labeling caused by unclear blood vessel boundaries. Also, we normalized the data. Image amplification methods, including translation, affine transformation and random noise, were adopted in this study. Random angular rotation was used to simulate small body twists of subjects during imaging examinations.

on methods, including translation, affine transformation and random noise, were adopted in this study. Random angular rotation was used to simulate small body twists of subjects during imaging examinations.

The whole network has four layers of downsampling and four layers of upsampling. Each layer in downsampling consists of two 3 × 3 × 3 convolutions, batch normalization, and max_pooling. There are two inputs in the upsampling process, one is from the next layer, and the other is the output from the same layer of downsampling. Splicing the two

inputs together ensures that the restored feature map incorporates features of different scales. The prediction accuracy of the DL models was checked on the validation dataset during the training process. We stopped our training when the prediction accuracy started to decrease to prevent overfitting. During the model training, the ADAM gradient descent optimization algorithm was adopted, with a batch size of 2, an initial learning rate of 0.0001 and 400 epochs. We used Python as the programming language. The software used was PyTorch 0.4.1, Python 3.6, Numpy, OpenCV and SimpleITK, and the hardware used was an NVIDIA Tesla P100 16G GPU for model training.

Python as the programming language. The software used was PyTorch 0.4.1, Python 3.6, Numpy, OpenCV and SimpleITK, and the hardware used was an NVIDIA Tesla P100 16G GPU for model training.

References:

(1) Xie T, Li Y, Lin Z, et al. Deep learning for fully automated segmentation and volumetry of Couinaud liver segments and future liver remnants shown with CT before major hepatectomy: a validation study of a predictive model. Quant Imaging Med Surg. 2023;13(5):3088-3103. doi:10.21037/qims-22-1008

(2) Tang A, Desai A, Hamilton G, et al. Accuracy of MR imaging-estimated proton density fat fraction for classification of dichotomized histologic steatosis grades in nonalcoholic fatty liver disease. Radiology. 2015;274(2):416-425. doi:10.1148/radiol.14140754
015;274(2):416-425. doi:10.1148/radiol.14140754
(3) Dixon M, Cruz J, Sarwani N, Gusani N. The Future Liver Remnant : Definition, Evaluation, and Management. Am Surg. 2021;87(2):276-286. doi:10.1177/0003134820951451.

(4) Çiçek Ö, Abdulkadir A, Lienkamp SS, Brox T, Ronneberger O. 3D U-Net: Learning Dense

Volumetric Segmentation from Sparse Annotation. In: Ourselin S, Joskowicz L, Sabuncu M, Unal G, Wells W, eds. Medical Image Computing and Computer-Assisted Intervention – MICCAI 2016.

MICCAI 2016. Lecture Notes in Computer Science, vol 9901. Cham, Switzerland: Springer, 2016;

424–432.
